# PE-Iv (Panorama Education-Italian version): the adaptation/validation of 5 scales, a step towards a SEL approach in Italian schools

**DOI:** 10.3389/fpsyg.2022.1026264

**Published:** 2022-12-01

**Authors:** Lynda S. Lattke, Aurelia De Lorenzo, Michele Settanni, Emanuela Rabaglietti

**Affiliations:** Department of Psychology, University of Turin, Turin, Italy

**Keywords:** SEL, school well-being, adaption/validation, measuring socio-emotional skills, adolescents

## Abstract

Implementing a Social and Emotional Learning (SEL) approach in school requires monitoring certain skills. As awareness of SEL increases in Italy, it is necessary to provide instruments to monitor these skills within a systemic approach. This study presents the cultural adaptation/validation of 5 scales from Panorama Education, which are widely used in school districts in the United States, to the Italian middle/high school context: Grit, Sense of Belonging, Self-Management, Social Awareness, and Self-Efficacy. After cultural adaptation, 709 middle/high school students answered an online questionnaire (2021). Psychometric properties showed good internal consistency and confirmatory factor analysis showed a good fit index. The differences in gender and grade level support the validity of the instrument.

## Introduction

Since the 1990s, social and emotional skills have been continuously studied in school contexts both in the United States (Elias et al., 1997; Jones and Bouffard, 2012; Kautz et al., 2021) and in Europe (Cottini and Morganti, 2015; Cefai et al., 2018). These skills are also known by many names worldwide: from non-cognitive skills, 21st Century Skills to non-academic skills and employability skills (Jones et al., 2019). In Italy, they are mainly taught under the umbrella of social and emotional education and social training (Cefai et al., 2018). Teaching and learning these skills in a systemic way within a school context is often referred to as social emotional learning (SEL) which benefits students’ academic performance and relationships (Payton et al., 2000; Jones and Bouffard, 2012). SEL combines theories from different models (Payton et al., 2000) which are mostly inspired on the social–emotional dimension such as Emotional Intelligence Theory (Goleman, 1995; Mayer and Salovey, 1997), Social Information Processing (Crick and Dodge, 1994) and cognitive/behavior change such as problem behavior theory (Jessor, 1991), and social cognitive theory (Bandura, 1998) amongst others. SEL is defined as how children and adults learn to understand and manage emotions, set goals, show empathy for others, build positive relationships, and make responsible decisions (Elias et al., 1997). When implemented as a whole-school approach, SEL can help children and youth and those in relationship with them improve their well-being (individually and as a class) by promoting academic achievement, problem-solving skills, social inclusion in school, and a reduction in bullying and antisocial behavior, leading to improved school climate (Greenberg et al., 2003; Durlak et al., 2011). In other words, promoting socio-relational skills can help improve students’ relationships with peers and teachers and increase their satisfaction with the school experience by increasing their academic engagement (Durlak et al., 2011), thereby reducing school dropout (Wang et al., 2016) especially after 2 years since the start of the pandemic which has profoundly changed the way we understand schooling (Lattke et al., 2020). Moreover, the earlier in age this approach is put into practice, the sooner children and youth are able to become more self/socially aware and self-determined (Davidow et al., 2016; Denham, 2018; De Lorenzo et al., 2021).

A SEL approach is part of a health promoting school (Turunen et al., 2017) which means it is necessary to engage the different components of the school community (School leadership, teachers, non-teaching staff, students and parents) in order to have a shared understanding of the importance of well-being and health (CASEL, 2017; Cefai et al., 2018; Bada et al., 2019; Velasco and Meroni, 2021). And, although measures to promote SEL are widespread at the European level, to our knowledge there are still few instruments in Italian schools which regularly monitor SE skills with a SEL approach particularly amongst the adolescent middle and high school populations, as is the case in many school districts in the United States (Kendziora and Yoder, 2016). For these reasons, we propose the cultural adaption/validation to the Italian context of 5 scales which measure some of these skills: Self-efficacy, Self-Management, Grit, Sense of Belonging and Social Awareness. As reported by a number of studies amongst which Durlak et al. (2010) and Cefai et al. (2018), these skills are associated amongst themselves. The scales we adapted were developed by Panorama Education, a US-based research organization founded in 2012 which assists schools in monitoring SEL. We believe their scales can be useful in the Italian context, where -as mentioned earlier- the awareness on the importance of these skills is growing but has not yet become systemic in the schools (Cefai et al., 2018).

In Italy, the Ministry of Education considers social–emotional skills (SE) as important as academic skills (MIUR, 2018). Some SE skills are included in social and civic competencies (i.e., autonomy, responsibility, cooperation, and readiness to learn) (European Communities, 2007). In educational environments where these skills are fostered and practiced, students advance from one grade level to the next, graduate from high school (Kautz et al., 2019), and have a better chance of becoming employable after graduation (Zins and Elias, 2007; Cefai et al., 2018). It is therefore incumbent upon schools to determine how to ensure that students achieve these educational, academic, and social goals by the end of each school cycle (MIUR, 2018). In spite of this, Italy is the fourth European country with the highest school dropout rate (OECD, 2019; De Luca et al., 2020; ISTAT, 2020; EUROSTAT, 2021) and with the largest “Not in Education, Not in Employment, Not in Training” population (NEET) on the continent (EUROSTAT, 2021). Based on a number of studies, if SEL is implemented as part of a whole-school approach, it can reduce dropout rates (Durlak et al., 2010; Downes, 2011; Montero-Sieburth and Turcatti, 2022). As a result, we could argue that a systemic SEL approach could be part of the solution, by strengthening students’ social and emotional skills while ensuring that they have the relational tools necessary to address some of life’s challenges and reduce social inequities, therefore resulting in an improved state of well-being (Freudenberg and Ruglis, 2007).

One of the most important components of a systemic SEL approach is monitoring, as it can help the school community understand how best to help its students acquire the skills necessary to address the various challenges students may face in life, whether on a personal or professional level (Jones and Bouffard, 2012; Duckworth and Yeager, 2015). To facilitate this process, we propose to consider scales developed by Gehlbach and his colleagues and which are among the most widely used in over 400 school districts in different US states (Panorama Education, 2016).

Panorama scales were developed based on the concept of SEL. Their scales are brief and use simple, clear, and easy-to-read language; they have been validated on samples of middle and high school students who come from geographic areas in the Southwest and Southeast of the United States, areas of the country known for high cultural and ethnic diversity (West et al., 2018a; RAND Corporation, 2019). Given the recent history of migration to Italy and the fact that part of the philosophy of SEL is to contribute to inclusion (Elias et al., 1997), this is an important element that could also help educators better understand the needs of youth from non-Italian backgrounds and create a more inclusive environment in which there is more openness to understanding these differences.

These scales are not divided by age but by grade level. For example, one set of questions is aimed at children in grades 3 to 5, while another is aimed at adolescents in grades 6 to 12. Our study focuses on the latter. Moreover, Panorama scales were developed in the age of digital natives, and it is hoped that the data obtained will help better capture the needs and perceptions of younger people. Although there are scales that measure the constructs we propose for the Italian school context, they are not part of a battery of instruments that measure SE skills within a SEL approach in school. Therefore, we expect that adapting these scales may help facilitate the implementation of a systemic SEL approach in Italy.

In this study we describe the cultural adaptation process and the evaluation of the psychometric properties of 5 of Panorama Education scales for which we performed inferential statistical analysis such as confirmatory and reliability analysis in order to adapt them to the Italian context.

## Materials and methods

### Sample and procedure

Our research team submitted a request to the Bioethics Committee of the University of Turin and subsequently obtained permission (Prot. No. 202854) to conduct the research on which this article is based. The research team then contacted various schools throughout the country, of which a total of three middle schools and eight high schools agreed to participate in our study. After the schools agreed to participate in the study, we sent a letter to the families and students explaining the purpose of the study, possible implications, and the time needed to complete the questionnaire (20 min). Finally, in order for students to participate in the online questionnaire, parents/guardians and students had to give their consent. A total of 709 students (27% from middle school and 73% from high school), mainly from northern and central Italy, completed a self-administered questionnaire in Spring 2021. The majority were female (75.2%) with a mean age of 15.44 years (SD =2.18; min = 11; max = 19).

### Instrument

Each of the scales used in this study was developed by Panorama Education (2016) specifically for middle and high school students. These scales can be found in the Panorama Social–Emotional Learning Survey User’s Guide (n.d.). The scales in this survey monitor student and teacher SE skills. For the purpose of this study, we focus only on 5 of these skills specifically for students. The guide includes “recommended” and “supplemental scales,” all 5 scales in this study are classified as “recommended.”

Below is a description of each of these 5 Likert scales which have a 5-point-score system:

*Grit (5 items)* measures “how well students are able to persevere through setbacks to achieve important long-term goals.” The answer options range from 1 to 5 with a minimum score of 5 to a maximum score of 25. The answer options range from ‘not at all’ to ‘always’ except for the last item in which the answer options range from ‘not at all likely’ to ‘very likely’.

*Sense of belonging (5 items)* measures “how much students feel they are valued members of the school community.” The answer options range from 1 to 5 with a minimum score of 5 to a maximum score of 25. There is one type of answer option which ranges from ‘not at all’ to ‘always’.

*Social Awareness (8 items)* measures “how well students consider and empathize with the perspectives of others.” The answer options range from 1 to 5 with a minimum score of 8 to a maximum score of 40. There is one type of answer option which ranges from ‘not at all’ to ‘always’.

*Self-efficacy (5 items)* measures “how much students believe they can be successful in achieving academic outcomes.” The answer options range from 1 to 5 with a minimum score of 5 to a maximum score of 25. There is one type of answer option which ranges from ‘not at all’ to ‘always’.

*Self-Management* (10 items) measures “how well students manage their emotions, thoughts, and behaviors in different situations.” The answer options range from 1 to 5 with a minimum score of 10 to a maximum score of 50. There is one type of answer option which ranges from ‘almost never’ to ‘almost always’.

### Validation measures

#### Participants’ demographic survey

The questionnaire also included a sociodemographic section in which participants were asked to provide information about their age, gender, and grade level.

### Qualitative and quantitative data analysis

The qualitative data analysis of the 5 original Panorama Education scales took place after culturally adapting them to the Italian language context; for this purpose we used the guidelines of Beaton et al. (2000). After data collection, the quantitative data analysis took place in two steps:

1. For all 33 original items of the 5 scales, we performed descriptive statistics, a reliability analysis (Cronbach’s Alpha) before structural validation, and a confirmatory factor analysis. The latter analysis resulted in a new version of 27 items of the 5 scales.
2. We then performed a new reliability analysis, a correlation analysis between the constructs and a *t*-test to observe any differences in terms of gender and grade level.

## Results

### Cultural adaptation

Cultural adaptation process according to Beaton et al. (2000) guidelines:

#### Forward translation

Panorama’s scales were first translated into Italian by three different native Italian speakers who teach in English at all levels of education, including middle and high school. Our research team then discussed the different versions and agreed on the version that was closest to the original version based on semantics and language comprehension.

#### Backward translation

The questionnaire was then back-translated into English by two different native English speakers who were not familiar with the original English version of the questionnaire or the constructs we were studying. The results were again discussed by our research team, this time by comparing the original version, the Italian versions, and the new English versions. Our criteria for discussion was based on the semantic and idiomatic meaning of the questions to ensure that both literal and cultural translation were taken into account. Once we decided on a final version, we pre-tested all 5 scales.

#### Pre-test

A total of 30 students were invited to the Department of Psychology of the University of Turin to respond to the adapted version of the online questionnaire. Before completing the questionnaire, students were asked to note the time it took to complete it and to point out any problems they encountered. Students were then invited to participate in a focus group to discuss the various scales and any difficulties with the language. A total of 3 separate focus groups were conducted online, each lasting approximately 2 h; each focus group consisted of 10 students, balanced by gender, ranging in age from 12 to 16 years old and from second year of middle school to second year of high school.

During the focus group, we discussed each item of the questionnaire with the students to ensure that the wording, meaning, and order of the various scales were clear, including the time it took to complete the questionnaire. On average, students indicated that it took them between 10′ and 15′ to complete.

#### Discussion of the results

A team of 7 researchers (one methodologist, one translator, one linguist, one teacher, one developmental psychologist professor and two researchers who are experts in school psychology), based on each person’s field of expertise, discussed the results of each focus group in order to bring together a final result which was useful in creating the final version of each scale. Throughout the process, we were in contact with researchers from Panorama Education, who were readily available to provide answers to questions we had as we adapted the scales, such as when the mode of instruction was changed to online learning due to the COVID-19 pandemic.

#### Final version

After completing the final version, we administered the questionnaire to high school and middle school students in April/May 2021. We then analyzed the data from the student responses. For this purpose, we performed descriptive statistics, confirmatory factor analysis to check whether all items worked in the Italian context, reliability test (Cronbach’s alpha), correlation analysis between all scales, and t-tests for gender and grade level.

### Descriptive statistics

The items were analyzed using descriptive statistics based on the data collected (mean and SD) as well as skewness and kurtosis (see Table 1).

**Table 1:** Descriptive statistics.

Factor	Items	Mean (*)	SD	Skewness	Kurtosis
Grit	GR_1	3.46	1.004	−0.450	−0.033
	GR_2	3.84	0.979	−0.654	−0.069
	GR_3	3.64	1.057	−0.678	−0.065
	GR_4	3.26	0.980	−0.241	−0.474
	GR_5	4.04	0.946	−0.797	0.114
Sense of belonging	SB_1	2.82	1.092	0.022	−0.766
	SB_2	2.92	1.098	−0.003	−0.863
	SB_3	3.84	1.043	.-861	0.187
	SB_4	3.00	1.052	−0.108	−0.707
	SB_5	3.39	1.264	−0.412	−0.889
Social awareness	SA_1	3.89	0.869	−0.635	0.246
	SA_2	4.07	0.908	−0.954	0.638
	SA_3	4.09	0.951	−1.162	1.333
	SA_4	3.40	0.822	−0.222	0.154
	SA_5	2.75	1.075	−0.017	−0.689
	SA_6	3.50	0.990	−0.319	−0.427
	SA_7	3.89	0.916	−0.556	−0.255
	SA_8	3.45	1.016	−0.492	−0.098
Self-management	SM_1	3.70	1.163	−0.670	−0.313
	SM_2	4.13	0.937	−0.961	0.394
	SM_3	3.29	1.228	−0.297	−0.781
	SM_4	3.22	0.974	−0.424	0.078
	SM_5	3.78	0.925	−0.620	0.317
	SM_6	3.05	1.195	−0.100	−0.783
	SM_7	3.93	1.039	−0.929	0.453
	SM_8	4.60	0.665	−1.647	2.317
	SM_9	4.41	0.736	−1.333	2.301
	SM_10	3.57	1.131	−0.566	−0.316
Self-efficacy	SE_1	4.01	1.010	−1.018	0.664
	SE_2	3.36	0.949	−0.597	0.267
	SE_3	3.42	0.874	−0.378	−0.137
	SE_4	3.36	0.983	−0.370	−0.206
	SE_5	3.01	0.999	−0.221	−0.740

(*) The range of the total scores for each of the items is from 1 to 5.

### Confirmatory factor analysis

To further investigate the psychometric value of Panorama Education’s scales in the Italian context, we examined the factorial structure of the scales by means of a confirmatory factor analysis (CFA) using Jamovi (version 1.6) statistical software. Following Kline (2015) the model fit was evaluated by using the following fit indexes (x^2^, CFI, TLI, RMSEA and SRMR). We first tested the fit of the data based on the dimensional model (Model 1) of 5 correlated dimensions according to one of the possible scale selection’s from the Panorama Social–Emotional Learning Survey. The fit indexes (x^2^ = 3,809, df = 495, CFI = 0.547, TLI = 0.517, RMSEA = 0.0940, SRMR = 0.08) were inadequate, furthermore, 6 items showed poor factor loadings (lower than 0.40; Table 2). The items were the following:

**Table 2:** Factor loadings.

Factor	Items	Estimate	SE	*Z*	*p*
Grit	GR_2	0.567	0.0409	13.86	< 0.001
	GR_3	0.582	0.0441	13.19	< 0.001
	GR_4	0.614	0.0408	15.06	< 0.001
	GR_5	0.495	0.0393	12.57	< 0.001
Sense of Belonging	SB_1	0.799	0.0372	21.51	< 0.001
	SB_2	0.419	0.0410	10.23	< 0.001
	SB_3	0.673	0.0371	18.15	< 0.001
	SB_4	0.854	0.0342	24.95	< 0.001
	SB_5	1.087	0.0403	27.00	< 0.001
Social awareness	SA_1	0.515	0.0367	14.02	< 0.001
	SA_2	0.471	0.0390	12.08	< 0.001
	SA_3	0.427	0.0411	10.40	< 0.001
	SA_6	0.557	0.0423	13.17	< 0.001
	SA_7	0.516	0.0400	12.90	< 0.001
	SA_8	0.499	0.0431	11.58	< 0.001
Self-management	SM_1	0.673	0.0434	15.51	< 0.001
	SM_2	0.609	0.0339	17.95	< 0.001
	SM_3	0.682	0.0453	15.03	< 0.001
	SM_4	0.657	0.0342	19.24	< 0.001
	SM_5	0.560	0.0341	16.42	< 0.001
	SM_7	0.384	0.0414	9.26	< 0.001
	SM_10	0.379	0.0462	8.19	< 0.001
Self-efficacy	SE_1	0.674	0.0357	18.85	< 0.001
	SE_2	0.693	0.0325	21.35	< 0.001
	SE_3	0.665	0.0293	22.65	< 0.001
	SE_4	0.774	0.0325	23.81	< 0.001
SE_5	0.523	0.0364	14.35	< 0.001

Grit #1: “How often do you stay focused on the same goal for several months at a time?”

Social Awareness #4: “How well did you get along with students who are different from you?”

Social Awareness #5: “How clearly were you able to describe your feelings?”

Self-Management #6: “How often did you remain calm, even when someone was bothering you or saying bad things?”

Self-Management #8: “How often were you polite to adults?”

Self-Management #9: “How often were you polite to other students?”

**Figure 1:**
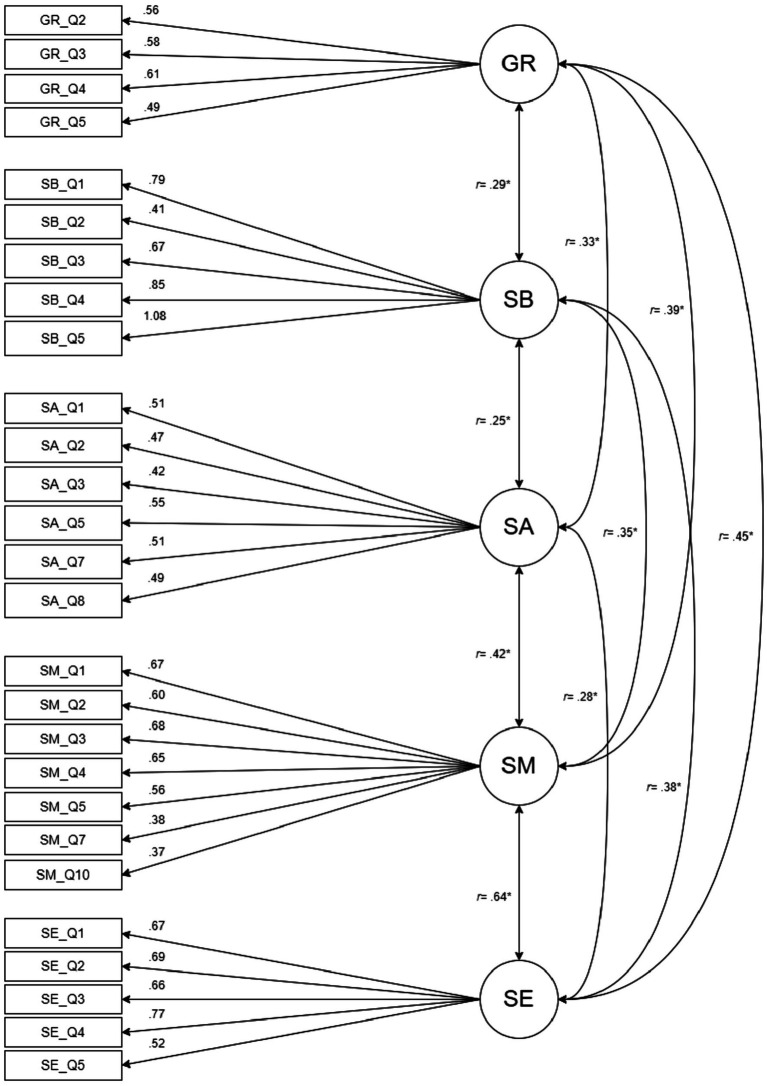
CFA with factor loadings from model 2 (5 factors, 27 items). Loadings are standardized. Rectangles indicate measured variables and circles represent latent constructs. Note the item numbering is retained from the original 33-item scale. *p < 0.001. GR, grit; SB, sense of belonging; SA, social awareness; SM, self-management; SE, self-efficacy.

As a result, the research team qualitatively assessed these items based on their relevance in measuring the constructs being tested. Since these items do not change the semantic properties of the constructs, we eliminated them based on the results of the CFA (Model 1). A new CFA (Model 2) was conducted, resulting in improved fit indexes (x^2^ = 590, df = 277, CFI = 0.948; TLI = 0.934; RMSEA = 0.0398, SRMR = 0.04). The resulting model is shown in Figure 1.

### Reliability statistics (Cronbach’s alpha)

After performing a CFA, we measured the internal consistency using Cronbach’s Alpha of each of the new scales. Based on De Vellis (2003), we can see that the values reported on Table 3 are good.

**Table 3:** Correlations between scales.

Scale	1	2	3	4	5	Alpha of Cronbach
Grit						0.659
Sense of belonging	0.292**					0.806
Social awareness	0.335**	0.252**				0.747
Self-management	0.394**	0.351**	0.428**			0.739
Self-efficacy	0.456**	0.389**	0.281**	0.642**		0.821

**p < 0.001.

### Spearman correlations between PE-Iv scales

Table 3 shows that the correlations between all constructs is positive. The associations with the highest values are between Self-Efficacy and Self-Management, between Self-Efficacy and Grit and between Self-Management and Social Awareness.

### Difference between groups

In order to investigate the validity of the criterion, differences in the means between males and females and between middle and high school students were tested. Means between gender groups (Table 4) were found to be significant especially for the Social Awareness [*t*(707) = 5.105, *p* < 0.001] and Self-Management [*t*(707) = 2.918, *p* < 0.05] scales for female participants. In the case of grade level (Table 5), we see instead that the Self-Efficacy [t(707) = 2.893, p < 0.05] and Sense of Belonging [*t*(707) = 2.750, *p* < 0.05] scales (which kept the same number of items as in Panorama Education) were significant for middle school students, while Social Awareness [*t*(707) = −3.513, *p* < 0.001] was significant for high school students.

**Table 4:** Difference between groups.

	Gender	*N*	Mean	*SD*
Grit	F	533	14.79	2.83
	M	176	14.76	2.62
Sense of belonging	F	533	15.92	4.24
	M	176	16.20	3.95
Social awareness	F	533	23.29	3.72
	M	176	21.65	3.57
Self-management	F	533	25.92	4.60
	M	176	24.76	4.48
Self-efficacy	F	533	17.11	3.78
	M	176	17.36	3.38

**Table 5:** Difference between groups based on grade level.

	Grade	*N*	Mean	*SD*
Grit	MS	192	14.85	2.73
	HS	517	14.76	2.80
Sense of belonging	MS	192	16.69	4.21
	HS	517	15.73	4.13
Social awareness	MS	192	22.07	4.00
	HS	517	23.18	3.61
Self-management	MS	192	25.49	4.93
	HS	517	25.68	4.46
Self-efficacy	MS	192	17.82	3.70
	HS	517	16.93	3.65

## Discussion

Monitoring socio-emotional skills is a concrete way for schools to meet the social–emotional needs of their students (Minnesota State Department of Education, 2019). Although preparing students with these skills is considered important by the Italian Ministry of Education (MIUR, 2018), there is currently no systemic SEL approach at school that includes regular monitoring of these skills. This study describes the process of the cultural adaptation of 5 scales (commonly used in middle and high schools in the United States) to the Italian context. These scales were tested in Italian middle (11–14 year-old students) and high schools (15–19 year-old students) and then the data were analyzed. As far as we know, this is the first time that Panorama Education scales have been translated into Italian and furthermore, it is the first time they have been used with middle and high school students in Europe. The Panorama Education scales are part of a systemic approach that is widely used in many American schools and are used as a monitoring tool in schools interested in applying a systemic SEL approach (Gehlbach and Hough, 2018; Kautz et al., 2019).

The study shows that the psychometric properties of the adapted scales are adequate: the instrument applied in the Italian context shows that these 5 scales correlate with each other as in the original version (Panorama Education, n.d.). Nevertheless some of these items showed a different behavior than expected, and for this reason they were eliminated. We can assume that one of the reasons why these items showed lower factor loadings in the confirmatory factor analysis is partly because their wording was interpreted differently by the Italian students who participated in the study; after these items were removed, the fit indices improved.

In general, most of the associations between the constructs for the two populations, American and Italian, show similarities, especially for the Sense of Belonging and for Grit and how these relate to other constructs. We also find that Self-Efficacy and Self-Management show the strongest association, an association that may have a positive impact on academic achievement, as reported in the results of the District of Columbia’s 2019 Panorama survey (DCPS, 2019) and other studies such as in Gehlbach and Hough (2018). These results represent evidence of construct validity of the instrument. On the other hand, however, some differences emerge, particularly in Social Awareness and its relationship to other constructs within our student population. Amongst these, the association between Sense of Belonging and Social Awareness is interesting, being stronger in the case of Panorama (Panorama Education, 2015, 2016). A possible explanation for this result is that these particular skills are not systematically promoted amongst students in Italian schools, nor is there a systemic approach in the school context, in spite of the existence of projects which do target the development of these skills (Giannotta and Weichold, 2016; Rabaglietti et al., 2021). In addition, the data were collected during the COVID-19 pandemic, an unusual period in which teaching had already taken place at a distance for an extended period of time, which had a particular impact on the relational side of high school students (Guazzini et al., 2022). With fewer interactions in the physical presence of each other, this may have resulted in an overall weaker relationship between Sense of Belonging and level of Social Awareness. Independent of the strength of each of the associations, several studies confirm the association between various SE constructs (Oriol et al., 2017; Malti et al., 2018; Kanopka et al., 2020; Vestad et al., 2021).

Criterion validity was studied by testing for differences in the means between males and females and between middle and high school students. In terms of gender, the Social Awareness and Self-Management scales had higher mean scores for females, which is confirmed by other studies that have used the same scales (Gehlbach and Hough, 2018; Kautz et al., 2021). Duckworth and Seligman (2006) also report higher levels of self-regulation in girls and how this contributes to their better academic performance compared to boys. Other studies confirm that Social Awareness is stronger in females (Mestre et al., 2009; Kågesten et al., 2016). Based on grade level, Social Awareness was greater for high school students, which is also confirmed by other studies (Gaspar et al., 2018; Van der Graaff et al., 2018; West et al., 2018b). It is interesting to note that Social Awareness continued to be important for high school students despite the distance learning context in which students had less interaction with their classmates; it could be argued that this situation actually increased their level of Social Awareness, but further studies are needed to confirm this. In contrast, at lower grade levels, Sense of Belonging and Self-Efficacy were more important. These findings are consistent with studies reporting that Self-Efficacy decreases as grade level increases; however, this may also depend on specific demographic factors such as socioeconomic, cultural, amongst others (West et al., 2018a). Sense of Belonging may have been higher among middle school Italian students because they were physically able to go to school compared to high school students, which meant they had the opportunity to form more meaningful relationships with their classmates.

Based on these findings, we could argue that mastery of these skills during adolescence requires that they be part of a systemic, whole-school approach in which each component of the school community plays an active role in promoting these skills. In addition, as noted earlier, it is important to keep in mind when reading our findings that they were collected during the COVID-19 pandemic, when distance education had been implemented for an extended period of time, portraying a different scenario compared to previous years (Grazzani et al., 2022; Paulus, 2022).

These adaptation/validation results suggest that these scales can be considered a stepping stone for monitoring students’ social–emotional skills in the Italian school context. However, as Gehlbach and Hough (2018) state, “validation is a process,” so we will continue to collect data to further examine these scales psychometrically to ensure that each scale measures the construct it is intended to measure and, more importantly, that they can be used in a systematic way in schools that wish to adopt a SEL approach.

## Conclusion

Our adaptation/validation of 5 scales from Panorama Education in the Italian context is intended as a first step toward measuring and monitoring social–emotional indicators as part of a systemic whole-school SEL approach. These scales can have a dual function at the international and national levels:

First, being a first adaptation of some of the Panorama Education scales in the European context, PE-Iv can contribute to the international discussion on the whole-school SEL approach. Second, specifically in the Italian context, PE-Iv can play a promotional role and motivate schools to adopt this approach.

This may raise awareness of the importance of SEL by relying on actual data, and hopefully, as mentioned earlier, be a step toward this systemic approach (CASEL, 2019; Meyers et al., 2019) which can help students improve academic performance and potentially reduce dropout rates (Kautz et al., 2021; Beccaria et al., 2022). However, this study has some limitations. Since these are new scales for the Italian context, it would have been useful to test the scales on the same population at two different time points. Furthermore, in a future study, we would like to test the scales with different populations and compare them with other scales that measure the same constructs (Kambara et al., 2021).

A whole-school SEL approach is a call to proactively seek ways to provide better opportunities for children and youth, especially in light of pandemic times and their impact on education, which increases the learning gap, especially among disadvantaged populations (Sormunen et al., 2022) including university students (Sulla et al., 2022). In addition, the SEL approach is a way to reduce inequities (Elias et al., 1997; Gehlbach and Hough, 2018; Allbright et al., 2019).

It is no longer enough for schools to teach content and subject-based instruction. As part of a systemic approach, the use of PE-Iv can extend the findings of the scientific literature on the importance of social–emotional competencies. By bringing a valuable monitoring tool into the classroom and into the daily lives of students, it will be possible to follow the development of social–emotional skills to support adolescents, their future challenges and their mental health.

## Data availability statement

The datasets generated for this study are available on request to the corresponding author.

## Ethics statement

The studies involving human participants were reviewed and approved by Università degli studi di Torino/Comitato di Bioetica dell’Ateneo. Written informed consent to participate in this study was provided by the participants’ legal guardian/next of kin.

## Author contributions

LSL and ER contributed to conceptualization, investigation, and formal analysis. MS supervised the final analysis. LSL, ADL, and ER contributed to writing – original draft. LSL, ADL, MS, and ER contributed to writing – review and editing. ER contributed to supervision. All authors contributed to the article and approved the submitted version.

## Funding

All the funding regarding the realization of this study was received internally to the authors’ organization. There was no additional external funding received for this study.

## Conflict of interest

The authors declare that the research was conducted in the absence of any commercial or financial relationships that could be construed as a potential conflict of interest.

## Publisher’s note

All claims expressed in this article are solely those of the authors and do not necessarily represent those of their affiliated organizations, or those of the publisher, the editors and the reviewers. Any product that may be evaluated in this article, or claim that may be made by its manufacturer, is not guaranteed or endorsed by the publisher.

## Supplementary material

The Supplementary material for this article can be found online at: https://www.frontiersin.org/articles/10.3389/fpsyg.2022.1026264/full#supplementary-material


