# Corrigendum: PE-Iv (Panorama Education-Italian version): the adaptation/validation of 5 scales, a step towards a SEL approach in Italian schools

**DOI:** 10.3389/fpsyg.2024.1541416

**Published:** 2025-01-08

**Authors:** Lynda S. Lattke, Aurelia De Lorenzo, Michele Settanni, Emanuela Rabaglietti

**Affiliations:** Department of Psychology, University of Turin, Turin, Italy

**Keywords:** SEL, school well-being, adaption/validation, measuring socio-emotional skills, adolescents

In the published article, the Supplementary material was mistakenly not included in the publication. The missing Supplementary material has been uploaded and published.

The authors apologize for this error and state that this does not change the scientific conclusions of the article in any way. The original article has been updated.

