# Supplementary material for: PE-Iv (Panorama Education-Italian version): the adaptation/validation of 5 scales, a step towards a SEL approach in Italian schools
Source: Front Psychol. 2022 Dec 1;13:1026264. doi: 10.3389/fpsyg.2022.1026264 (PMC9751788; doi:10.3389/fpsyg.2022.1026264)
Supplement: Supplementary file 1 [file Data_Sheet_1.pdf]

Appendix

Italian version of 5 scales from Panorama Education

Grit (Grinta)

Scegli la risposta più adatta a te:

1. Se non riesci a raggiungere un obiettivo per te importante, quanto è probabile che ci riprovi?

2. Quando stai lavorando su un progetto che significa molto per te, riesci a mantenere la concentrazione se ci sono molte distrazioni?

3. Se hai un problema mentre stai lavorando per un obiettivo importante, quanto riesci a continuare per bene il lavoro?

4. Ci sono alcune persone che riescono a lavorare su un loro obiettivo per molto tempo e altri che invece cambiano spesso i loro obiettivi:

A tuo parere, tra gli obiettivi che hai adesso, quanto è probabile che ce ne sarà almeno uno su cui continuerai a lavorare per i prossimi anni? (Panorama Education item #5)

Sense of Belonging (Senso di Appartenenza)

Di seguito trovi domande su come ti senti all'interno della tua classe ADESSO

1. Quanto, le persone nella tua classe, riescono a capire bene la persona che sei?
2. Quanto ti senti affezionato/a agli insegnanti della tua classe?
3. Quanto sono rispettosi verso di te gli studenti/le studentesse della tua classe?
4. Quanto sei importante per gli altri della tua classe?
5. In generale, quanto ti senti parte/ senti di appartenere alla tua classe?

Social awareness (Consapevolezza Sociale)

Queste domande si riferiscono agli ULTIMI 30 GIORNI e ci aiutano a capire le tue relazioni con gli altri:

1. Quanto attentamente hai ascoltato il punto di vista di altre persone?
2. In generale, quanto hai tenuto conto dei sentimenti di altre persone?
3. Quanto spesso fai i complimenti alle persone che ottengono qualcosa di bello?
4. Quando altri non erano d'accordo con te, quanto sei riuscito/a a rispettare il loro punto di vista? (Panorama Education item #6)
5. In che misura sei riuscito/a a mantenere il tuo punto di vista senza mancare di rispetto agli altri? (Panorama Education item #7)
6. Fino a che punto sei riuscito/a ad essere in disaccordo con gli altri, senza iniziare a litigare? (Panorama Education item #8)

Self-Management (Auto-regolazione)

Le seguenti domande riguardano i tuoi comportamenti e i tuoi atteggiamenti a scuola durante gli ULTIMI 30 GIORNI, scegli la risposta più adatta a te:

Quanto spesso…….

1. …ti è capitato di arrivare alle tue lezioni preparato/a per una possibile interrogazione?
2. …hai seguito le indicazioni durante le lezioni quando sono state assegnate?
3. ….ti è capitato di fare i tuoi compiti subito invece di aspettare fino all’ultimo minuto?
4. …sei stato/a attento/a senza distrarti?
5. …sei rimasto/a concentrato/a, quelle volte che hai lavorato da solo/a?
6. …hai permesso che gli altri finissero di parlare senza interromperli? (Panorama Education item #7)
7. …sei riuscito/a a tenere sotto controllo la rabbia? (Panorama Education item #10)

Self-Efficacy (Auto-Efficacia)

Quanto spesso ti capita di pensare le cose che seguono?

1. Quanto pensi di riuscire a portare a termine tutti i compiti che ti sono stati dati durante le lezioni?
2. Quando a lezione vengono spiegati concetti difficili, quanto pensi di riuscire a capirli?
3. Quanto pensi di riuscire a imparare tutto quello che insegnano durante le lezioni?
4. Quanto pensi di riuscire a fare i compiti più difficili assegnati durante le lezioni?
5. Quanto pensi di ricordare l’anno prossimo quello che hai imparato a lezione quest’anno?
